# Low LCAT activity is linked to acute decompensated heart failure and mortality in patients with CKD

**DOI:** 10.1016/j.jlr.2024.100624

**Published:** 2024-08-20

**Authors:** Julia T. Stadler, Thomas Bärnthaler, Andrea Borenich, Insa E. Emrich, Hansjörg Habisch, Alankrita Rani, Michael Holzer, Tobias Madl, Gunnar H. Heine, Gunther Marsche

**Affiliations:** 1 Division of Pharmacology, Otto Loewi Research Center for Vascular Biology, Immunology and Inflammation, Medical University of Graz, Graz, Austria; 2 Institute for Medical Informatics, Statistics and Documentation, Medical University of Graz, Graz, Austria; 3 Saarland University, Faculty of Medicine, Homburg/Saarbrücken, Germany; 4 Division of Medical Chemistry, Otto Loewi Research Center for Vascular Biology, Immunology and Inflammation, Medical University of Graz, Graz, Austria; 5 BioTechMed Graz, Graz, Austria; 6 Department of Nephrology, Agaplesion Markus Krankenhaus, Frankfurt am Main, Germany

**Keywords:** LCAT, kidney, lipoproteins, atherosclerosis, CVD, cholesterol/metabolism, chronic kidney disease, survival, HDL

## Abstract

Chronic kidney disease (CKD) is often associated with decreased activity of lecithin-cholesterol acyltransferase (LCAT), an enzyme essential for HDL maturation. This reduction in LCAT activity may potentially contribute to an increased risk of cardiovascular mortality in patients with CKD. The objective of this study was to investigate the association between LCAT activity in patients with CKD and the risk of adverse outcomes. We measured serum LCAT activity and characterized lipoprotein profiles using nuclear magnetic resonance spectroscopy in 453 non-dialysis CKD patients from the CARE FOR HOMe study. LCAT activity correlated directly with smaller HDL particle size, a type of HDL potentially linked to greater cardiovascular protection. Over a mean follow-up of 5.0 ± 2.2 years, baseline LCAT activity was inversely associated with risk of death (standardized HR 0.62, 95% CI 0.50–0.76; *P* < 0.001) and acute decompensated heart failure (ADHF) (standardized HR 0.67, 95% CI 0.52–0.85; *P* = 0.001). These associations remained significant even after adjusting for other risk factors. Interestingly, LCAT activity was not associated with the incidence of atherosclerotic cardiovascular events or kidney function decline during the follow-up. To conclude, our findings demonstrate that low LCAT activity is independently associated with all-cause mortality and ADHF in patients with CKD, and is directly linked to smaller, potentially more protective HDL subclasses.

## Graphical abstract

**Figure:**
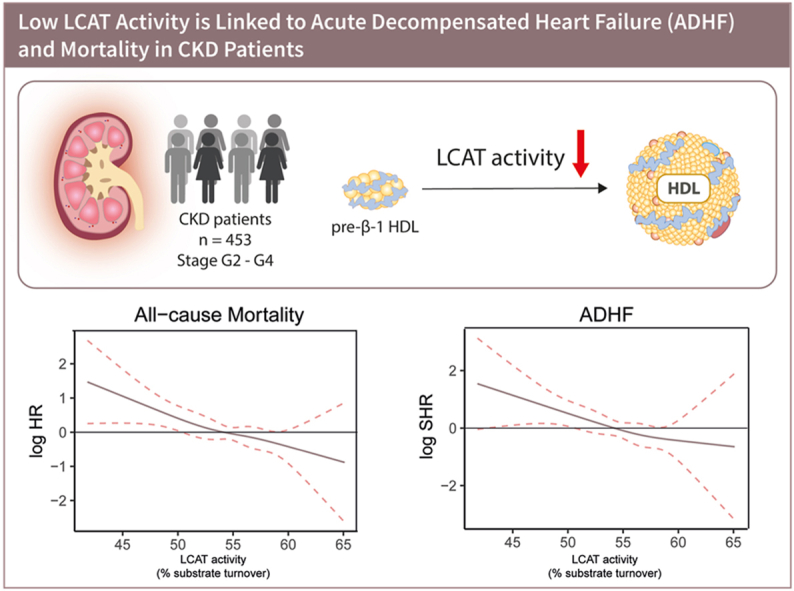

Chronic kidney disease (CKD) represents a pervasive and progressive health condition with a substantial impact on public health, given its rising prevalence, which is estimated to be approximately 13% globally (1, 2). Notably, the majority of affected people face extra-renal comorbidities, particularly cardiovascular complications, including coronary artery disease, heart failure, arrhythmia, and sudden cardiac death (3, 4, 5). Since CKD has emerged as one of the leading causes of mortality worldwide, further therapies to reduce mortality risk are urgently needed (2).

CKD is associated with several alterations in lipid metabolism, leading to atherogenic dyslipidemia, comprised of elevated plasma levels of triglycerides and low high-density lipoprotein (HDL)-cholesterol levels (6, 7). Impaired renal function directly affects the metabolism, composition, and functionality of HDL particles (8). Down-regulation of hepatic lecithin-cholesterol acyltransferase (LCAT) expression occurs in chronic renal failure (9). Compared to the general population, low LCAT activity in CKD severely delays the conversion of pre-β-1 HDL to α-migrating HDL (10, 11, 12, 13). LCAT is activated by apolipoprotein A-I and esterifies free cholesterol to cholesteryl-esters, causing the esterified cholesterol to transition from the particle's surface to its hydrophobic core. As a result, larger spherical alpha-HDL particles are formed, which transport cholesterol to the liver and steroidogenic tissues (14, 15). By transferring a fatty acid from phospholipids, LCAT generates lysophosphatidylcholine (LPC) by this process. Lipoprotein-associated LPCs are increasingly recognized as key players in the regulation of vascular inflammation in its various stages. They can exert both protective and deleterious effects, depending on the specific disease context (16).

Despite the hypothesized anti-atherogenic functions of LCAT, its role in disease progression remains complex. Mutations leading to fish-eye disease or LCAT deficiency exhibit discordant outcomes, with increased or even decreased carotid atherosclerosis, respectively (17). This suggests potential context-dependent adverse effects based on LCAT's ability to esterify cholesterol and also on apolipoprotein B-containing lipoproteins.

More recent research has shown that recombinant LCAT effectively corrects abnormal lipoprotein profiles in LCAT knockout mice (18), and clinical phase II trials are currently underway to evaluate its potential for treating familial LCAT deficiency (https://clinicaltrials.gov/study/NCT04737720).

The objective of this study was to investigate the potential association between LCAT activity and adverse clinical outcomes in patients with CKD. We aimed to clarify the intricate relationship between modified LCAT function, HDL composition, and cardiovascular complications in the context of CKD by measuring LCAT activity and utilizing NMR measurements for comprehensive lipoprotein profiling.

## Materials and methods

### Study design and patients

The CARE FOR HOMe (Cardiovascular and Renal Outcome in CKD 2–4 Patients – The Fourth Homburg Evaluation) study, a prospective cohort study, initially enrolled 526 patients with CKD stages G2-G4 based on the estimated glomerular filtration rate (eGFR) at baseline. Among these, 453 patients were included in our analysis, selected based on the availability of a suitable amount of serum material for conducting NMR measurements. The study intended to investigate risk factors for CKD progression and to identify patients at high risk for cardiovascular complications. Patients were all treated in the renal outpatient clinic of the Saarland University Medical Centre in Homburg, Germany. The study excluded patients with acute kidney injury, transplant recipients, pregnant women, and participants under 18 years of age. Further, patients receiving systemic immunosuppressive medications and patients with apparent infections were excluded. At baseline, fasted blood samples and a spot urine sample of the participants were obtained. To collect data about the medical history, current medication, smoking status, and prevalence of diabetes mellitus, a standardized questionnaire was used. In addition, twenty age-matched healthy subjects with a median eGFR of 86 (77, 94) were included (21–523 ex 09/10), and their clinical characteristics are presented in supplemental Table S2. The study was conducted following the Declaration of Helsinki and approved by the local Ethics Committee. All patients gave their written informed consent.

### Outcome analysis

Annually, all patients were invited to the study center for a follow-up examination, in which information on cardiovascular events was collected by a standardized questionnaire. Patients who became dialysis-dependent during follow-up and patients who refused to attend the regular follow-up visit were contacted by telephone, and laboratory information was provided by their treating primary care physician or their nephrologist. Two independent physicians adjudicated all events; in case of disagreement, a third physician was consulted.

The study endpoints included several critical health outcomes: all-cause mortality, hospital admission for acute decompensated heart failure (ADHF)—defined as admission for a clinical syndrome involving symptoms (progressive dyspnea) in conjunction with clinical (peripheral edema or pulmonary rales), and/or radiologic (cardiomegaly, pulmonary edema, or pleural effusions) signs. Endpoints also included atherosclerotic cardiovascular events (ASCVE), defined as the first occurrence of any of the following: myocardial infarction, coronary artery angioplasty/stenting/bypass surgery, major stroke, carotid endarterectomy/stenting, nontraumatic lower extremity amputation, and/or lower limb artery angioplasty/stenting/bypass surgery and cardiovascular death. In addition, deterioration of renal function, characterized by a reduction in estimated glomerular filtration rate (eGFR) of more than 50% and initiation of renal replacement therapy, was assessed.

### Serum LCAT activity

LCAT activity in serum was evaluated using a commercially available kit from Merck following the provided manufacturer's instructions. This assay measures the phospholipase activity of LCAT. In detail, serum samples were subjected to a 4-h incubation at 37°C with the LCAT substrate. The fluorescent substrate emitted fluorescence at 470 nm, and upon hydrolysis by LCAT, a monomer was released, emitting fluorescence at 390 nm. The assessment of LCAT activity was performed over time and expressed as the change in 470/390 nm emission intensity.

### Preβ-1 HDL

The concentration of serum pre-β-1 HDL was determined using a commercially available enzyme-linked immunosorbent assay kit from Immbiomed, following the protocol provided by the manufacturer.

### NMR spectroscopy measurements

Blood serum samples for NMR spectroscopy analysis were prepared as described previously (19, 20). NMR spectra were recorded on a Bruker 600 MHz Avance Neo NMR spectrometer (Bruker). Data analysis for lipoprotein quantification was carried out using the Bruker IVDr Lipoprotein Subclass Analysis (B.I.LISATM) method. This is conducted by sending raw NMR spectrum data to a Bruker online server, which returns 112 lipoprotein parameters (e.g., free and total cholesterol, triglyceride, and total phospholipid content of main classes and subclasses of VLDL, IDL, LDL, and HDL, as well as ApoA-I, ApoA-II, and ApoB protein concentrations). The Bruker IVDr Lipoprotein Subclass Analysis provides 4 HDL subclasses (HDL-1 to HDL-4 sorted according to increasing density and decreasing size, respectively). The subclasses of HDL are defined as follows: HDL-1 ranges from 1.063 to 1.100 kg/L, HDL-2 ranges from 1.100 to 1.112 kg/L, HDL-3 ranges from 1.112 to 1.125 kg/L, and HDL-4 ranges from 1.125 to 1.210 kg/L. For simplicity, we have labeled the subclasses as L-HDL (HDL-1), M-HDL (HDL-2), S-HDL (HDL-3), and XS-HDL (HDL-4).

### Statistical analyses

Patients were stratified according to tertiles of LCAT activity followed by comparing several clinical parameters between the groups. Characteristics were summarized using mean (SD) for normally distributed variables and median (Q1, Q3) for variables with skewed distributions. Categorical variables are presented as number of patients (percentage). Differences between the groups were calculated using one-way ANOVA, the Kruskal-Wallis test, and Pearson’s Chi-squared test. Kaplan-Meier plots for all-cause mortality and cumulative incidence plots for other outcomes across LCAT tertiles, followed by log-rank tests, were used to assess the association of LCAT with adverse clinical outcomes in CKD. Cox regression models were further used with standardized LCAT activity and hazard ratios were expressed per increase of 1 standard deviation for the endpoint all-cause mortality. Competing risk analyses were performed for the outcomes ADHF, ASCVE, and renal decline. Restricted cubic spline analyses were performed to visualize the adjusted association of continuous LCAT activity with the risk of adverse outcomes. Correlations between LCAT activity and HDL-related parameters derived from NMR measurements were calculated using Spearman correlation coefficients. A *P*-value < 0.05 was considered significant. Statistical analyses were performed using GraphPad Prism 10.0.0, SPSS Statistics 26, and R version 4.3.2.

## Results

This study included a total of 453 patients with CKD, of which sufficient serum material for LCAT activity and NMR measurements was available. This research constitutes a secondary analysis of the observational CARE for HOMe study, which originally enrolled pre-dialysis CKD patients in stages 2–4 (Stage2, n = 97; Stage 3a, n = 147; Stage 3b, n = 126; Stage 4, n = 83). The clinical characteristics of patients stratified in CKD stages are shown in supplemental Table S1. The primary focus was on investigating risk factors contributing to the progression of cardiovascular and renal outcomes (21, 22, 23). The baseline clinical characteristics of study participants stratified by serum LCAT activity are shown in Table 1. The mean age of patients at baseline was 65 (12) years, 39% of patients were female, and the majority were overweight or obese, with a median body mass index of 30.1 (26.8, 33.4). Among patients, 39% had been diagnosed with diabetes mellitus and 33% had prevalent cardiovascular disease. The age among patients significantly differed across LCAT activity tertiles, with higher age noted in tertile 1. CKD patients further differed in BMI, statin medication, glutamic-oxaloacetic transaminase (GOT), and diastolic blood pressure levels. Although tertiles of LCAT did not display any differences in eGFR or occurrence of micro-or macroalbuminuria, other markers of renal function including creatinine clearance (CrCl) and cystatin C were significantly altered. No differences were noted in CRP levels, total cholesterol, LDL-cholesterol, HDL-cholesterol, and pre-β-1 HDL across tertiles, while serum triglyceride levels differed significantly. Levels of the cardiac biomarker NT-proBNP, the left atrial volume index (LAVI), and the mean carotid intima-media thickness (IMT) were higher in individuals within the lowest tertile of LCAT activity. The prevalence of hypertension was similar across all tertiles, with a significant difference in the use of anti-hypertensive medication observed only for thiazide diuretics.

**Table 1:** Baseline clinical characteristics of the study cohort stratified by tertiles of serum LCAT activity

Parameter	Tertile 1 N = 151	Tertile 2 N = 152	Tertile 3 N = 150	Overall N = 453	*P*-value
LCAT activity (% substrate turnover)	51.3 (48.9–52.6)	55.2 (54.2–55.8)	58.2 (57.3–59.6)	55.2 (52.6–57.3)	**<0.001**^1^
Age (y)	67 (12)	65 (12)	63 (12)	65 (12)	**0.015**^2^
Female sex (n, %)	64 (42.4%)	58 (38.2%)	56 (37.3%)	178 (39.3%)	0.629^3^
BMI (kg/m^2^)	28.5 (25.2, 32.1)	30.5 (27.1, 33.6)	31.5 (27.9, 34.2)	30.1 (26.8, 33.4)	**<0.001**^1^
Creatinine eGFR (ml/min/m^2^)	45 (31, 57)	47 (37, 57)	47 (34, 59)	47 (34, 58)	0.357^1^
CRP (mg/l)	2.5 (1.0, 4.9)	2.3 (1.2, 5.1)	3.0 (1.5, 5.5)	2.7 (1.2, 5.1)	0.148^1^
Hba1c (%)	5.9 (5.5, 6.4)	5.6 (5.4, 6.4)	6.0 (5.5, 6.4)	5.8 (5.5, 6.4)	0.281^1^
GOT (U/l)	24 (21, 29)	25 (22, 32)	27 (23, 31)	26 (22, 31)	**0.010**^1^
Prevalent CVD (n, %)	47 (31.1%)	55 (36.2%)	46 (30.7%)	148 (32.7%)	0.524^3^
Diabetes Mellitus (n, %)	56 (37.1%)	54 (35.5%)	67 (44.7%)	177 (39.1%)	0.220^3^
Total cholesterol (mg/dl)	196 (179, 223)	188 (160, 219)	197 (165, 229)	192 (165, 224)	0.577^1^
HDL-cholesterol (mg/dl)	54 (45, 67)	51 (45, 60)	50 (45, 57)	51 (45, 61)	0.091^1^
LDL-cholesterol (mg/dl)	94 (75, 118)	87 (73, 116)	93 (71, 111)	92 (72, 114)	0.410^1^
Triglycerides (mg/dl)	137 (102, 189)	148 (108, 199)	162 (118, 237)	148 (108, 209)	**0.016**^1^
Pre-β-1 HDL (mg/dl)	5.2 (3.9, 7.0)	5.4 (4.0, 7.0)	5.3 (4.0, 74)	5.3 (4.0, 7.0)	0.887^1^
NT-proBNP (ρg/ml)	328 (106, 900)	209 (86, 671)	145 (61, 293)	208 (88, 543)	**<0.001**^1^
LAVI (ml/m^2^)	39.3 (30.4, 50.0)	34.2 (29.5, 48.4)	33.2 (27.4, 39.4)	35.0 (29.3, 44.9)	**<0.001**^1^
Mean carotid artery IMT (mm)	0.71 (0.62, 0.83)	0.68 (0.58, 0.80)	0.65 (0.58, 0.76)	0.68 (0.60, 0.80)	**0.020**^1^
CrCl (ml/min)	50 (34, 73)	60 (43, 81)	63 (44, 84)	59 (41, 79)	**0.002**^1^
Cystatin C (mg/l)	1.6 (1.3, 2.4)	1.5 (1.2, 2.0)	1.4 (1.2, 1.8)	1.5 (1.2, 2.0)	**0.001**^1^
Cystatin C eGFR (ml/min/m^2^)	39 (23, 52)	44 (31, 57)	50 (34, 63)	44 (28, 57)	**<0.001**^2^
Microalbuminuria (n, %)	48 (32.0%)	43 (28.3%)	43 (28.7%)	134 (29.6%)	0.740^3^
Macroalbuminuria (n, %)	35 (23.3%)	29 (19.1%)	34 (22.7%)	98 (21.7%)	0.627^3^
Current nicotine (n, %)	19 (12.6%)	18 (11.8%)	12 (8.00%)	49 (10.8%)	0.389^3^
Statins	58 (38.4%)	84 (55.3%)	94 (62.7%)	236 (52.1%)	**<0.001**^3^
Other lipid-lowering drugs	16 (10.6%)	17 (11.2%)	21 (14.0%)	54 (11.9%)	0.622^3^
Systolic BP (mmHg)	148 (134, 164)	150 (137, 167)	151 (141, 168)	150 (137, 167)	0.276^1^
Diastolic BP (mmHg)	83 (74, 92)	87 (77, 94)	87 (78, 97)	85 (76, 94)	**0.017**^1^
Hypertension (n, %)	104 (69.3%)	113 (74.8%)	116 (77.3%)	333 (73.8%)	0.272^3^
Nitrates (n, %)	11 (7.3%)	9 (5.9%)	13 (8.7%)	33 (7.3%)	0.656^3^
Diuretics (n, %)	118 (78.1%)	126 (82.9%)	124 (82.7%)	368 (81.2%)	0.491^3^
Loop diuretics (n, %)	68 (45.3%)	75 (49.3%)	56 (37.3%)	199 (44.0%)	0.102^3^
Thiazide diuretics (n, %)	68 (45.0%)	82 (53.9%)	89 (59.3%)	239 (52.8%)	**0.043**^3^
Aldosterone inhibitors (n, %)	26 (17.2%)	37 (24.5%)	34 (22.7%)	97 (21.5%)	0.277^3^
β-Blockers (n, %)	102 (67.5%)	104 (68.4%)	112 (74.7%)	318 (70.2%)	0.338^3^
ACE inhibitors/angiotensin receptor blockers (n, %)	115 (76.2%)	127 (83.6%)	124 (82.7%)	366 (80.8%)	0.204^3^
Monoxidin/Clonidine (n, %)	26 (17.2%)	31 (20.4%)	31 (20.7%)	88 (19.4%)	0.702^3^
Calcium channel blockers (n, %)	71 (47.0%)	80 (52.6%)	83 (55.3%)	234 (51.7%)	0.338^3^
Peripheral α-Blocker (n, %)	17 (11.3%)	19 (12.5%)	14 (9.3%)	50 (11.0%)	0.676^3^
Other hypertensives (n, %)	9 (6.0%)	7 (4.6%)	7 (4.7%)	23 (5.1%)	0.832^3^

Differences between the groups were calculated with ^1^Kruskal-Wallis test, ^2^One-way ANOVA, or ^3^Pearson’s Chi-squared test. Values for categorical variables are given as numbers (percentages) and values for continuous variables are given as median (Q1–Q3). Significant results are highlighted in bold.

BMI, body mass index; BP, blood pressure; CrCl, creatinine clearance; CRP, C-reactive protein; CVD, cardiovascular disease; eGFR, estimated glomerular filtration rate; GOT, glutamic-oxaloacetic transaminase.

Twenty age-matched healthy subjects with a median eGFR of 86 (77, 94) were included, and their clinical characteristics are presented in supplemental Table S2.

### LCAT activity is associated with adverse outcomes in patients with CKD

Patients were stratified by eGFR categories, calculated using the CKD-EPI equation based on serum creatinine levels. Compared to control patients with normal renal function, those with CKD stages G3a, G3b, and G4 exhibited reduced LCAT activity levels (supplemental Fig. S1A). No significant differences in LCAT activity were observed among the various CKD stages. Additionally, we calculated eGFR using the CKD-EPI equation based on serum cystatin C levels. This analysis revealed significantly lower LCAT activity in patients with advanced CKD, with notable differences observed between patients in stages G4/5 and those in stages G1/2 and G3a (see supplemental Fig. S1B).

During a mean follow-up of 5.0 ± 2.2 years, 19% of patients died of any cause, 12% had to be hospitalized due to ADHF, 23% had an incidence of ASCVE, and 16% of total patients reached the renal endpoint, defined as reduction of eGFR by 50% and initiation of renal replacement therapy.

Measurements of serum LCAT activity in the CARE for HOMe study indicated significant differences between patients who deceased during the follow-up period and those who survived (supplemental Fig. S1C).

Furthermore, patients who had to be hospitalized for ADHF exhibited diminished serum LCAT activity, whereas no significant difference was observed in patients experiencing either the ASCVE or renal decline (supplemental Fig. S1D–F).

To explore the relationship between LCAT activity and adverse outcomes in CKD patients, participants were categorized into tertiles based on serum LCAT levels. Kaplan-Meier survival analyses were conducted to assess mortality, while cumulative incidence curves were generated to evaluate other time-to-event outcomes. These analyses allowed for the comparison of outcomes across LCAT tertiles and the identification of potential associations.

**Fig. 1:**
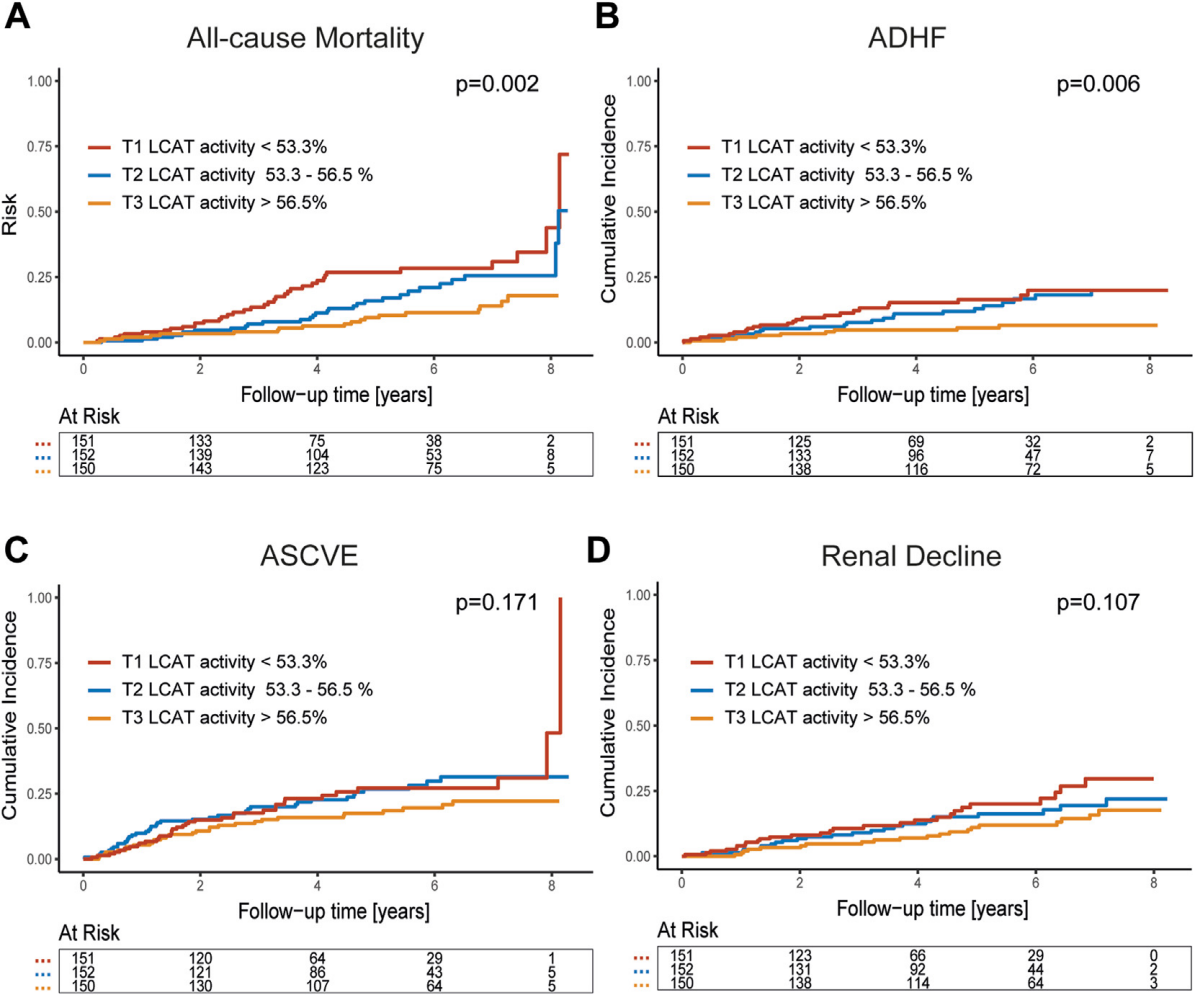
Risk of adverse outcomes among CKD patients and tertiles of LCAT activity. Kaplan-Meier and cumulative incidence plots with subsequent log-rank tests for risk of all-cause mortality (A), ADHF (acute decompensated heart failure) (B), the incidence of atherosclerotic cardiovascular events (C), or renal decline (D) are shown. The number of patients at risk at each time point is presented below the graph.

Of particular interest, subsequent log-rank tests revealed that patients with the lowest tertile of LCAT activity at baseline exhibited a higher mortality risk (*P* = 0.002) (Fig. 1A). No significant differences between tertiles of LCAT activity with the incidence of ASCVE were observed (Fig. 1C). Additional cumulative incidence analyses of other outcomes unveiled that low LCAT activity at baseline was associated with an increased risk for ADHF (*P* = 0.006) (Fig. 1B), while no difference between LCAT activity tertiles for renal decline (*P* = 0.107) was observed (Fig. 1D).

**Fig. 2:**
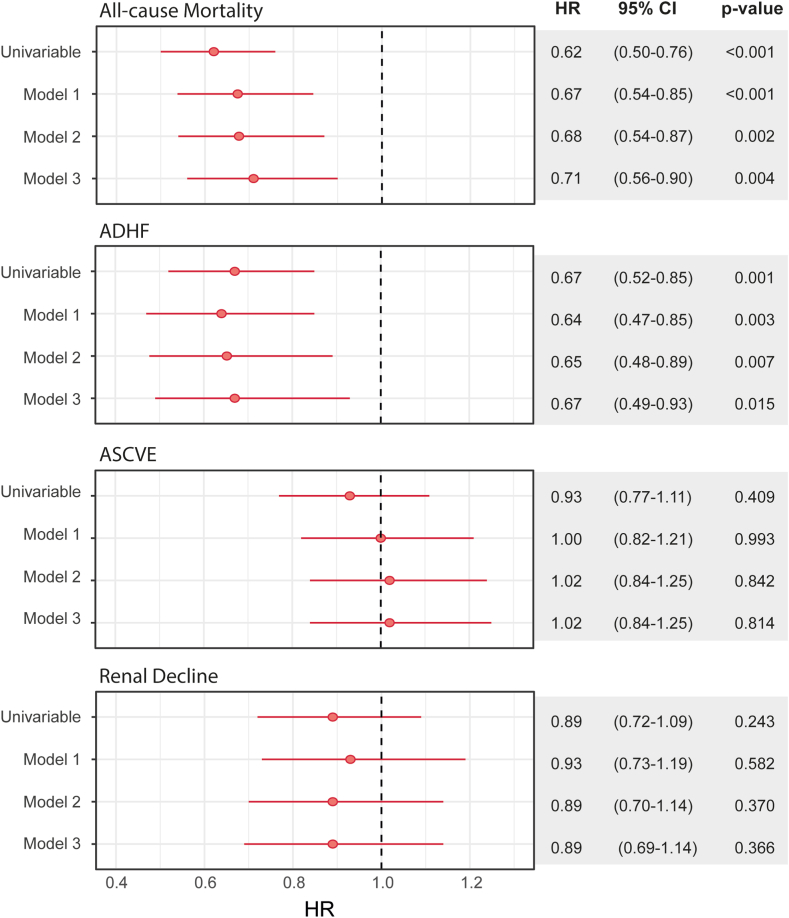
Hazard ratios per 1 SD increase and 95% confidence intervals (CI) from Cox regression and Competing Risk analyses. LCAT activity was used as a continuous variable and association with risk of all-cause mortality, acute decompensated heart failure (ADHF), atherosclerotic cardiovascular events (ASCVE), and renal decline was assessed. Model 1 is adjusted for age, sex, BMI, and eGFR; Model 2 is Model 1 additionally adjusted for prevalent CVD, blood pressure, current smoking, log-transferred CRP, diabetes mellitus, statins or other lipid-lowering medication, and total cholesterol. Model 3 is Model 2 additionally adjusted for LDL-C, HDL-C, hypertension, and log-transferred albuminuria, while total cholesterol had to be removed from the model due to multicollinearity.

Cox proportional hazard analyses demonstrated that LCAT activity, considered as a continuous variable, was inversely associated with risk of all-cause mortality (standardized HR, 0.62 [95% CI, 0.50–0.76]; *P* < 0.001) (Fig. 2). Moreover, univariable competing risk analyses also demonstrated a significant association of LCAT activity with ADHF (standardized HR, 0.67 [95% CI, 0.52–0.85]; *P* = 0.001), while no significant association was observed with the risk of ASCVE or renal decline. After adjustment for traditional cardiovascular and renal risk factors (Model 2), LCAT activity remained significantly associated with risk of all-cause mortality (standardized HR, 0.68 [95% CI, 0.54–0.87]; *P* = 0.002) and ADHF (standardized HR, 0.65 [95% CI, 0.48–0.89]; *P* = 0.007). After further adjustments for LDL-cholesterol, HDL-cholesterol, the prevalence of hypertension, and albuminuria, the associations for LCAT activity with both all-cause mortality and ADHF remained significant. Given the observed differences in thiazide diuretic use across LCAT activity tertiles, we added this medication to Model 2 (supplemental Table S3, Model 4). This adjustment still revealed a significant inverse association between LCAT activity and both mortality (standardized HR, 0.69 [95% CI, 0.54–0.87]; *P* = 0.003) and ADHF (standardized HR, 0.66 [95% CI, 0.48–0.90]; *P* = 0.009).

**Fig. 3:**
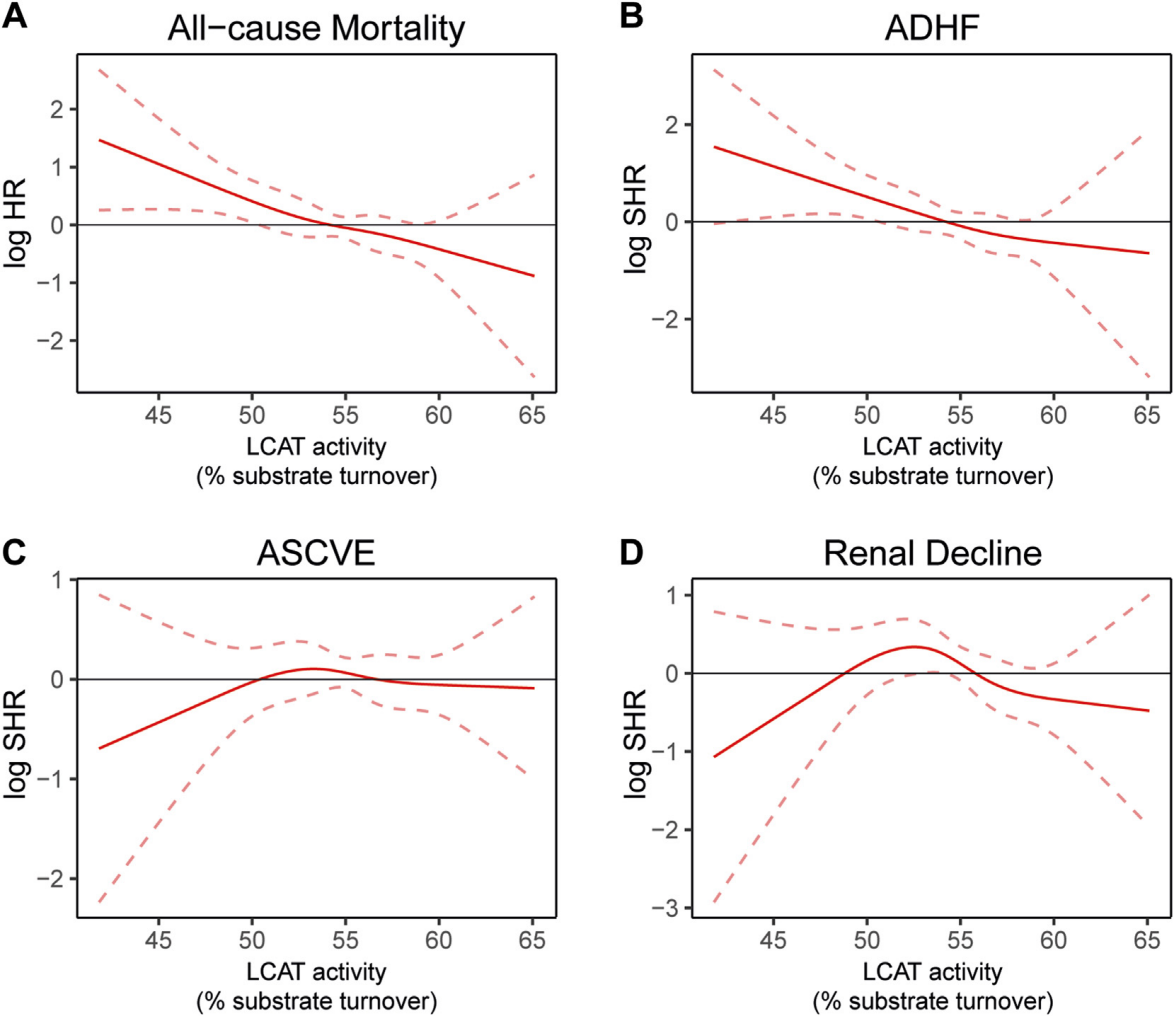
Visualization of the association between LCAT activity and adverse outcomes in CKD using restricted cubic spline plots. For endpoint all-cause mortality (A) Cox regression analyses were performed, while for ADHF (B), ASCVE (C), and renal decline (D), competing risk analyses were conducted, resulting in subdistributional hazard ratios. The model was adjusted for age, sex, BMI, eGFR, systolic blood pressure, diastolic blood pressure, current smoking, prevalent CVD, diabetes mellitus, log-transferred CRP, and statin or other lipid-lowering medication, LDL-C, HDL-C, hypertension, and log-transferred albuminuria. Solid lines indicate log-transferred (subdistributional) hazard ratios and dashed lines indicate a 95% confidence interval. ADHF, acute decompensated heart failure; ASCVE, atherosclerotic cardiovascular event.

To investigate potential non-linear relationships of LCAT activity with the endpoints, we computed cubic spline plots from Cox regression and competing risk analyses of Model 3 (Fig. 3). Similarly, we observed an association of low serum LCAT activity with increased risk for all-cause mortality and ADHF (Fig. 3A, B).

### Association of LCAT with HDL subclasses and composition

**Fig. 4:**
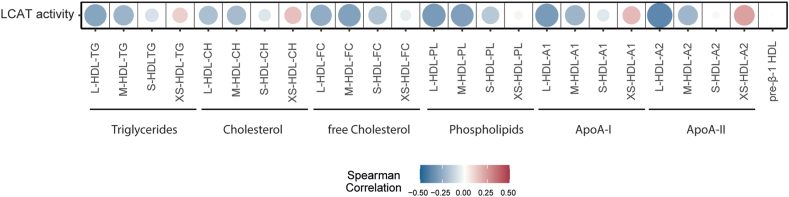
Correlations between LCAT activity with NMR-measured HDL parameters. Each cell of the heatmap represents a pairwise Spearman correlation between the two parameters indicated in the respective row and column. The circles' size and colour indicate the correlation's strength and direction. Subclasses are defined as follows: L-HDL: 1.063–1.100 kg/L; M-HDL: 1.100–1.112 kg/L; S-HDL: 1.112–1.125 kg/L; XS-HDL: 1.125–1.210 kg/L; HDL-subclasses: L, large; M, medium; S, small; XS, extra small.

We performed correlation analyses to evaluate the relationship between LCAT activity with lipoprotein parameters from NMR measurement (Fig. 4). We observed a significant positive correlation between LCAT activity and the smallest XS-HDL subclass composition, including XS-HDL triglycerides, cholesterol, phospholipids, apoA-I, and most prominently apoA-II. Furthermore, a robust negative correlation was noted between LCAT activity and parameters of large and medium-sized HDL subclasses, while the association with small HDL was weaker. Despite the increased serum pre-β-1 HDL in advanced CKD (supplemental Fig. S2), no association of LCAT activity with pre-β-1 HDL was observed. LCAT deficiency is associated with low levels of both HDL-cholesterol and LDL-cholesterol. However, in our study, we found no correlation between LCAT activity and LDL-cholesterol levels (r_S_ = −0.046, *P* = 0.326).

## Discussion

Treatment strategies that focus solely on traditional atherosclerotic risk factors appear to be less effective in reducing the rate of cardiovascular events in patients with CKD than in the general population (24, 25). Hence, there is an unmet medical need in the field of cardiorenal medicine to identify novel targets for therapeutic intervention.

Decreased LCAT activity is consistently reported in patients with CKD (10, 11, 12, 13). The purpose of the present study was to evaluate the association between serum LCAT activity and adverse outcomes in patients with CKD. The results showed that low LCAT activity was associated with a higher risk of all-cause mortality and ADHF, independent of traditional cardiovascular and renal risk factors. NMR-based lipoprotein profiling revealed direct correlations between LCAT activity and compositional parameters of XS-HDL.

It is well established that LCAT activity is decreased in CKD (10, 11, 12, 13). A more recent study reported an association between LCAT protein concentration and renal impairment in patients with CKD (26). In good agreement, our initial analyses examining LCAT activity in patients across various stages of CKD revealed a significant reduction in serum LCAT activity compared to control patients with normal renal function. Furthermore, using the CKD-EPI equation based on serum cystatin C levels, we observed a further reduction in LCAT activity with advanced CKD. Moreover, when patients were stratified into tertiles based on their LCAT activity levels, notable differences in creatinine clearance and cystatin C levels were observed, indicating a potential link between LCAT activity and renal function. Additionally, in the tertile with the lowest LCAT activity, levels of the cardiac biomarker NT-proBNP, as well as left atrial volume index and mean carotid artery intima-media thickness were higher. These findings imply a potential association between cardiac function and LCAT activity. It is important to note that we evaluated LCAT activity rather than LCAT serum protein concentrations. This distinction is crucial because LCAT activity can be significantly affected by the uremic environment, inflammatory state, and oxidative stress present in CKD. Specifically, LCAT is vulnerable to direct oxidative inactivation of its sulfhydryl groups, resulting in steric hindrance and impaired activity (27). Uremic toxins that accumulate in CKD are associated with profound changes in HDL lipid and protein concentration (8). Specifically, there is a reduction in proteins such as apoA-I, apoA-II, and apoM, while proteins associated with inflammation, including serum amyloid A and apoC-III, are elevated (28, 29). CKD is further associated with carbamylation of apolipoproteins due to increased myeloperoxidase and urea-derived cyanate (30), and elevated levels of α-oxoaldehydes can covalently modify apoA-I altering the conformation of specific domains crucial for LCAT activation (31). The majority of our study cohort was overweight or obese, which is not surprising given the strong association with comorbidities such as type 2 diabetes and hypertension, both closely linked to kidney damage (2, 32). A significant difference in BMI was observed across the LCAT tertiles, with higher BMI values in patients exhibiting higher LCAT activity. This finding aligns with previous results suggesting that obese patients tend to have higher LCAT activity (33). While no differences in cholesterol levels were observed across LCAT tertiles, patients with higher LCAT activity exhibited higher triglyceride levels. These levels remained within the normal range, as our study included patients with early to moderate CKD. Severe dyslipidemia, characterized by elevated triglycerides and decreased HDL-C, typically occurs in more advanced CKD, such as stage 5 and end-stage renal disease (34).

Our study found no significant association between LCAT activity and ASCVD in this cohort. This observation is consistent with previous studies showing a lack of association between HDL-related factors, such as HDL cholesterol, HDL particle number, HDL cholesterol efflux capacity, and ASCVD in patients with CKD (21, 35). In addition, LCAT activity did not correlate with LDL cholesterol levels in our study, suggesting a possible additional explanation for the lack of association (36).

Notably, recent research suggests that LCAT plays a crucial role in the formation of small HDL particles (37, 38). Consistent with this, we observed direct correlations between LCAT activity and various compositional parameters of the XS-HDL subclass, but negative correlations with the large and medium-sized HDL subclasses.

LCAT activity plays a crucial role by providing substrates for CETP (39), catalyzing the rapid transfer of cholesteryl-esters from HDL to LDL or VLDL in exchange for triglycerides. Esterification of cholesterol by LCAT proceeds more slowly than the transfer of cholesteryl-esters by CETP, representing a rate-limiting step (40, 41). This might explain the observed negative correlation between LCAT activity and large HDL particles in the highest LCAT activity tertile in our study. Consistent with this assumption, we have observed in previous studies that LCAT phospholipase activity is positively correlated with small HDL subclasses and negatively associated with large HDL subclasses (33, 42). Serum pre-β-1 HDL levels were increased in more advanced CKD in our study, similar to previous studies (10, 12). Previous research has established that LCAT-dependent conversion is a critical factor in determining plasma pre-β-1 HDL concentration in healthy individuals (13). However, our study found no correlation between LCAT activity and pre-β-1 HDL levels in patients with CKD. This finding aligns with a prior study (10) and suggests that LCAT is not the sole determinant of pre-β-1 HDL in this patient population. Given the kidney's established role in HDL homeostasis, it is plausible that impaired renal catabolism of immature HDL particles significantly contributes to the elevated pre-β-1 HDL observed in CKD patients.

Beyond its core function of esterifying cholesterol, LCAT exhibits additional activities with potential antioxidant and anti-inflammatory implications. Studies have shown that LCAT can neutralize pro-inflammatory and pro-oxidant molecules such as platelet-activating factors and oxidized phospholipids with long chains at the sn-2 position (43, 44, 45). Our findings suggest that LCAT's role in modifying HDL particles is critical for their anti-atherogenic properties and could serve as a novel therapeutic target. Recently, recombinant LCAT has been shown to correct the abnormal lipoprotein phenotype in LCAT knockout mice (https://clinicaltrials.gov/study/NCT04737720). Therapies that restore LCAT function (protein and gene replacement therapies and LCAT activators) showed promising effects on markers of LCAT activity (46). A novel LCAT activator (DS-8190a) was shown to prevent the progression of plaque accumulation in atherosclerosis models (47).

Of particular interest, a single bolus of reconstituted HDL delivered after ischemia rapidly increased myocardial glucose uptake and improved structural remodeling in association with enhanced cardiac functional recovery in mice (48). Intriguingly, investigating how LCAT might contribute to these protective effects opens an exciting avenue for future research.

Another potential anti-atherogenic mechanism of LCAT should not be underestimated. Emerging evidence suggests that lysophosphatidylcholine (LPC), generated by LCAT from phospholipids, plays a previously underappreciated role in various diseases (16). Recent studies have shown an intriguing association between lower plasma LPC levels and worse clinical outcomes (16), particularly in CKD (49). LPC displays a wide range of beneficial biological activities, including inhibiting platelet aggregation and promoting blood vessel relaxation, which all may contribute to the potential protective effects of LCAT on the cardiovascular system (50, 51).

Importantly, extra-renal comorbidities including coronary artery disease, heart failure, arrhythmias, and sudden cardiac death are a leading cause of death in CKD patients (5), accounting for 30–40% of deaths (52). Given the current lack of therapies proven to reduce morbidity and mortality in patients with heart failure and CKD, our results support the hypothesis that pharmacological modulation of LCAT strengthens the case for potential treatment for ADHF in patients with CKD.

### Limitations and strengths

It is important to acknowledge the limitations of this study. The moderate sample size (n = 453) may have restricted our power to detect subtle associations, particularly given the heterogeneous nature of CKD. Moreover, the observational study design precludes definitive causal inferences. Despite these limitations, our findings of no association between LCAT activity and ASCVE underscore the need for further investigation to elucidate the complex role of LCAT in cardiovascular disease.

A key strength of this study is the prospective evaluation of all primary and secondary endpoints. The comprehensive baseline clinical characterization also allowed for robust adjustment of potential confounders.

## Conclusion

While the association between reduced LCAT activity and renal disease is well-established, our study introduces a novel perspective by linking LCAT activity to adverse outcomes across the entire CKD spectrum. Although our observational design prevents definitive causal claims, these findings strongly suggest that LCAT activators merit exploration as potential therapeutic targets for heart failure in patients with CKD.

## Data availability

All relevant data for this study are included in this manuscript. The data which were not shown are available upon request from the corresponding author.

## Supplemental data

This article contains supplemental data.

## Conflict of interest

The authors declare that they have no conflicts of interest with the contents of this article.
