# Supplementary material for: Low LCAT activity is linked to acute decompensated heart failure and mortality in patients with CKD
Source: J Lipid Res. 2024 Aug 20;65(9):100624. doi: 10.1016/j.jlr.2024.100624 (PMC11416249; doi:10.1016/j.jlr.2024.100624)
Supplement: supplemental Figs. S1 and S2, and Tables S1–S3 [file mmc1.docx]

**
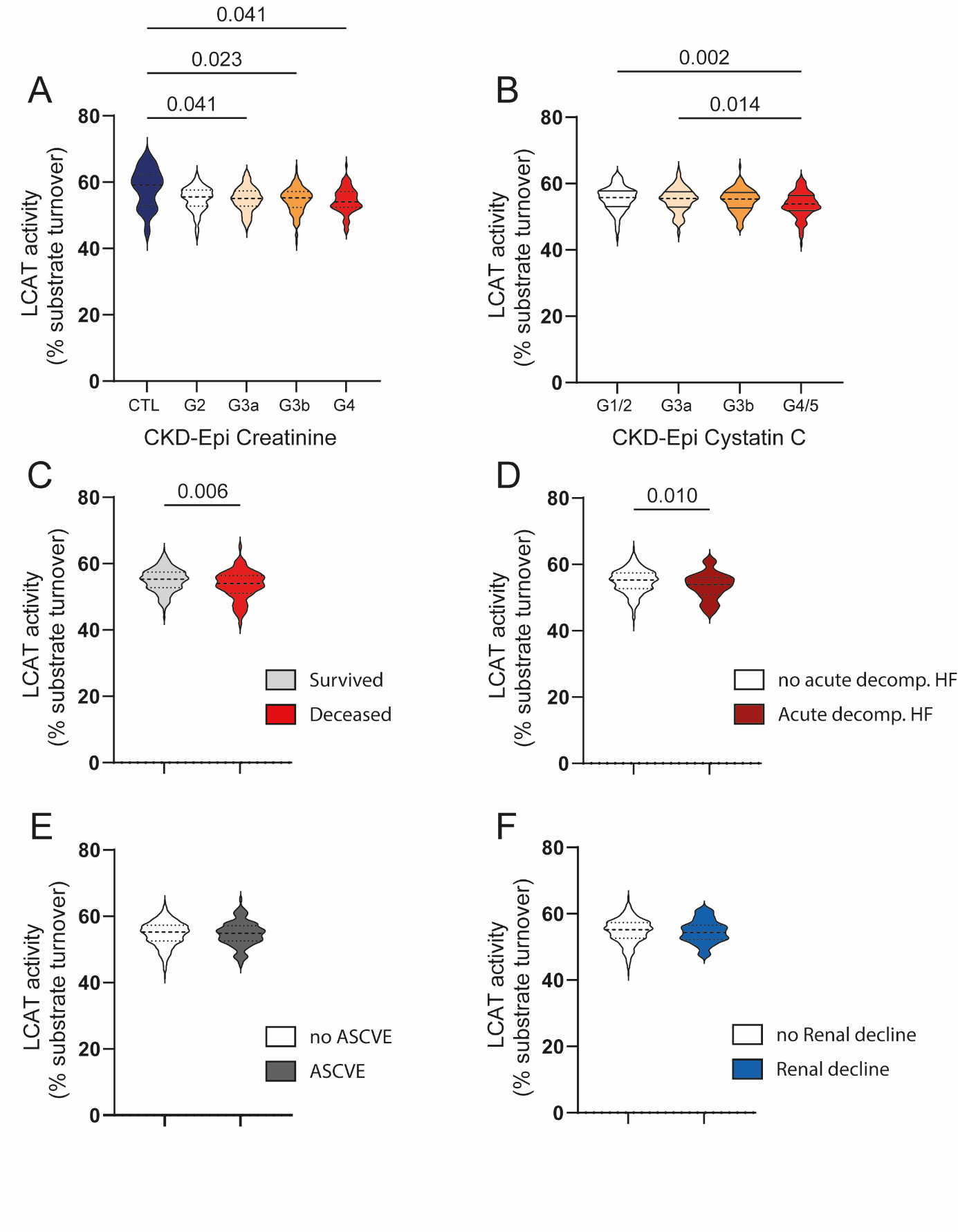
Supplementary Material:**

**Suppl. Figure 1.** Serum LCAT activity in CKD patients. Categorization of patients by eGFR category, estimated using (A) the CKD-EPI Creatinine equation and (B) the CKD-EPI Cystatin C equation. The patients were categorized based on survival status (C), by the occurrence of acute decompensated HF (D), by the incidence of an ASCVE (E), and by the occurrence of renal decline, defined as eGFR reduction of > 50% (F) during the follow-up period. HF, heart failure, ASCVE, acute decompensated heart failure.


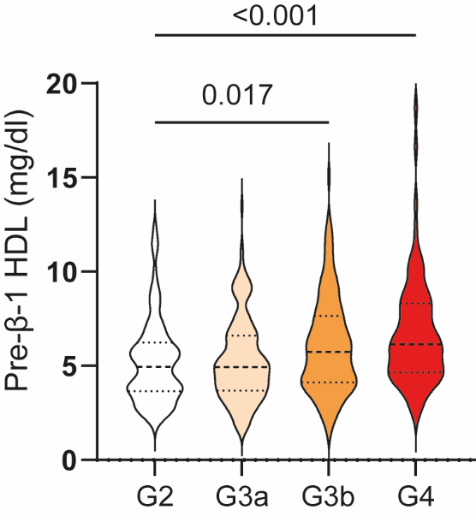


**Suppl. Figure 2.** Serum levels of pre-β-1 HDL in CKD patients stratified by eGFR category.

**Suppl. Table 1.** Baseline characteristics of patients stratified by creatinine eGFR category

| **Characteristic** | **G 2**,  N = 97 | **G 3 a**,  N = 147 | **G 3 b**,  N = 126 | **G 4**,  N = 83 | **Overall**,  N = 453 | **p-value** |
| --- | --- | --- | --- | --- | --- | --- |
| **Age (y)** | 59 (12) | 65 (13) | 68 (11) | 67 (12) | 65 (12) | **<0.001***^2^* |
| **Female sex** | 30 (30.9%) | 62 (42.2%) | 56 (44.4%) | 30 (36.1%) | 178 (39.3%) | 0.164*^1^* |
| **BMI (kg/m2)** | 29.6 (26.3,33.5) | 30.5 (27.0,33.7) | 30.3 (26.5,33.2) | 29.0 (26.7,32.9) | 30.1 (26.8,33.4) | 0.555*^3^* |
| **eGFR (ml/min/m2)** | 67 (63,72) | 52 (48,55) | 37 (34,41) | 23 (19,26) | 47 (34,58) | **<0.001***^3^* |
| **CrCl (ml/min)** | 95 (76,108) | 65 (57,80) | 46 (40,56) | 31 (23,37) | 59 (41,79) | **<0.001***^3^* |
| **Cystatin C (mg/l)** | 1.13 (0.99,1.22) | 1.34 (1.18,1.50) | 1.70 (1.51,2.06) | 2.45 (2.23,2.80) | 1.48 (1.21,2.03) | **<0.001***^3^* |
| **CRP (mg/l)** | 2.3 (1.2, 4.1) | 2.6 (1.1, 4.9) | 2.7 (1.2, 6.1) | 3.3 (1.1, 5.6) | 2.7 (1.1, 5.1) | 0.322^3^ |
| **Hba1c (%)** | 5.60 (5.40,6.00) | 5.70 (5.50,6.33) | 6.05 (5.53,6.75) | 6.00 (5.60,6.33) | 5.80 (5.50,6.40) | 0.512*^3^* |
| **GOT (U/l)** | 26 (22,32) | 26 (23,31) | 26 (22,31) | 24 (19,28) | 26 (22,31) | **0.046***^3^* |
| **Prevalent CVD** | 17 (17.5%) | 47 (32.0%) | 56 (44.4%) | 28 (33.7%) | 148 (32.7%) | **<0.001***^1^* |
| **Diabetes Mellitus** | 33 (34.0%) | 57 (38.8%) | 49 (38.9%) | 38 (45.8%) | 177 (39.1%) | 0.454*^1^* |
| **Current nicotine** | 18 (18.6%) | 13 (8.84%) | 11 (8.73%) | 7 (8.43%) | 49 (10.8%) | 0.053*^1^* |
| **Statins** | 38 (39.2%) | 81 (55.1%) | 74 (58.7%) | 43 (51.8%) | 236 (52.1%) | **0.026***^1^* |
| **Other lipid-lowering drugs** | 14 (14.4%) | 15 (10.2%) | 18 (14.3%) | 7 (8.43%) | 54 (11.9%) | 0.453*^1^* |
| **Systolic BP (mmHg)** | 145 (136,159) | 153 (140,170) | 146 (135,166) | 152 (141,166) | 150 (137,167) | **0.047***^3^* |
| **Diastolic BP (mmHg)** | 86 (79,96) | 88 (79,96) | 81 (74,91) | 85 (74,94) | 85 (76,94) | **0.002***^3^* |
| **Total cholesterol (mg/dl)** | 202 (179,230) | 193 (164,226) | 187 (163,215) | 187 (160,218) | 192 (165,224) | 0.122*^3^* |
| **HDL-cholesterol (mg/dl)** | 53 (45,62) | 51 (45,62) | 52 (45,61) | 49 (41,59) | 51 (45,61) | 0.194*^3^* |
| **LDL-cholesterol (mg/dl)** | 100 (79,121) | 92 (75,110) | 89 (72,106) | 87 (66,111) | 92 (72,114) | **0.024***^3^* |
| **Triglycerides (mg/dl)** | 146 (103,212) | 147 (105,203) | 148 (112,192) | 155 (119,218) | 148 (108,209) | 0.676*^3^* |
| **Hypertension (n, %)** | 65 (67.7%) | 117 (79.6%) | 84 (67.2%) | 67 (80.7%) | 333 (73.8%) | **0.026***^3^* |
| **Anti-hypertensive drugs (n, %)** | 92 (94.8%) | 139 (94.6%) | 123 (97.6%) | 83 (100.0%) | 437 (96.5%) | 0.090^1^ |

Clinical characteristics of CKD patients at baseline. Differences between the groups were calculated with ^1^Pearson’s Chi-squared test, ^2^One-way ANOVA or ^3^Kruskal-Wallis test. Values for categorical variables are given as numbers (percentages) and values for continuous variables are given as median (Q1-Q3). BMI, body mass index; eGFR, estimated glomerular filtration rate; CrCl, creatinine clearance; CRP, C-reactive protein; GOT, glutamic oxaloacetic transaminase; CVD, cardiovascular disease; BP, blood pressure.

**Suppl. Table 2.** Clinical characteristics of the control cohort.

| Characteristic | Controls (n=20) |
| --- | --- |
| LCAT activity (% substrate turnover) | 59.1 (53.8,62.2) |
| Age (years) | 65 (7) |
| Female Sex (n, %) | 11 (55.0%) |
| Diabetes (n, %) | 0 (0%) |
| Hypertension (n, %) | 10 (50.0%) |
| CRP (mg/l) | 0.90 (0.50,2.90) |
| Creatinine eGFR (ml/min/m^2^) | 86 (77,94) |

Values for categorical variables are given as numbers (percentages) and values for continuous variables are given as median (Q1-Q3).

**Suppl. Table 3.** Hazard ratios per 1 SD increase of LCAT activity and 95% confidence intervals (CI) from Cox regression and Competing Risk analyses

|  | All-cause mortality | | ADHF | | ASCVE | | Renal Decline | |
| --- | --- | --- | --- | --- | --- | --- | --- | --- |
| Parameter | **HR (95% CI)**  **Per 1 SD** | ***p*-value** | **HR (95% CI)**  **Per 1 SD** | ***p-*value** | **HR (95% CI)**  **Per 1 SD** | ***p-*value** | **HR (95% CI)**  **Per 1 SD** | ***p-*value** |
| Model 4 | 0.69 (0.54-0.87) | 0.003 | 0.66 (0.48-0.90) | 0.009 | 1.00 (0.82-1.22) | 0.994 | 0.91 (0.72-1.16) | 0.462 |

LCAT activity was used as a continuous variable to assess its association with the risk of all-cause mortality, acute decompensated heart failure (ADHF), atherosclerotic cardiovascular events (ASCVE), and renal decline. Model 4 includes the variables from Model 2 (adjusted for age, sex, BMI, eGFR, prevalent CVD, blood pressure, current smoking, log-transferred CRP, diabetes mellitus, and use of statins or other lipid-lowering medications, and total cholesterol), with an additional adjustment for thiazide diuretic medication.
